# Review on the Impact of Polyols on the Properties of Bio-Based Polyesters

**DOI:** 10.3390/polym12122969

**Published:** 2020-12-12

**Authors:** Kening Lang, Regina J. Sánchez-Leija, Richard A. Gross, Robert J. Linhardt

**Affiliations:** 1 Department of Chemistry and Chemical Biology and Center for Biotechnology and Interdisciplinary Studies, Rensselaer Polytechnic Institute, Troy, NY 12180, USA; langk2@rpi.edu (K.L.); regisanchez@uchicago.edu (R.J.S.-L.); 2 Pritzker School of Molecular Engineering, The University of Chicago, 5640 S Ellis Ave, Chicago, IL 60637, USA; 3 Department of Biomedical Engineering and Center for Biotechnology and Interdisciplinary Studies, Rensselaer Polytechnic Institute, Troy, NY 12180, USA; 4 Department of Biology and Center for Biotechnology and Interdisciplinary Studies, Rensselaer Polytechnic Institute, Troy, NY 12180, USA

**Keywords:** polyol, polyol polyesters, hyperbranched polymers, structural tailored-cut biopolymers, physical properties

## Abstract

Bio-based polyol polyesters are biodegradable elastomers having potential utility in soft tissue engineering. This class of polymers can serve a wide range of biomedical applications. Materials based on these polymers are inherently susceptible to degradation during the period of implantation. Factors that influence the physicochemical properties of polyol polyesters might be useful in achieving a balance between durability and biodegradability. The characterization of these polyol polyesters, together with recent comparative studies involving creative synthesis, mechanical testing, and degradation, have revealed many of their molecular-level differences. The impact of the polyol component on the properties of these bio-based polyesters and the optimal reaction conditions for their synthesis are only now beginning to be resolved. This review describes our current understanding of polyol polyester structural properties as well as a discussion of the more commonly used polyol monomers.

## 1. Introduction

The demand for biodegradable polymers in medical uses, such as the delivery of therapeutic drugs and tissue engineering, has inspired scholars in the material research community to develop new synthetic polymers. Relative to metals and ceramics, synthetic biodegradable polymers provide extra flexibility that can be adjusted to optimize tissue response, biodegradability, biocompatibility, and physical properties. In the past few decades, significant progress has been made in the design, synthesis, and use of biodegradable polymers for biomedical applications, such as drug delivery and tissue engineering [1,2,3,4]. The widespread application of polyesters as biodegradable polymers can be attributed to their many advantages [5,6].

Many examples of the use of polyester scaffolds as thermoplastics and thermosetting plastics are described in the literature [7,8]. A problem encountered with semicrystalline thermoplastic materials is the presence of crystalline regions that possess both crystalline and amorphous domains. This heterogeneity in morphology can lead to mechanical strength losses that are non-linear [9]. However, cross-linking of thermoset materials often leads to decreased crystallinity or fully amorphous materials with linear degradation rates and predictable mechanical strength loss. Furthermore, thermoset polyesters can offer enhanced performance in combination with improved thermal stability, chemical resistance, and structural integrity [10].

Polyol polyester polymers made from renewable resources, such as vegetable oils, sorbitol, diacids, and cellulose, can be used to reduce the high demand for petrochemical products and their harmful effects on the environment. They contribute to a large number of applications such as elastomers, sealants, and adhesives, to name a few. They are generally environmentally degradable and, during prolonged contact with tissues, polyol polyesters hydrolyze to the natural building blocks they were built from [7].

**Figure 1:**
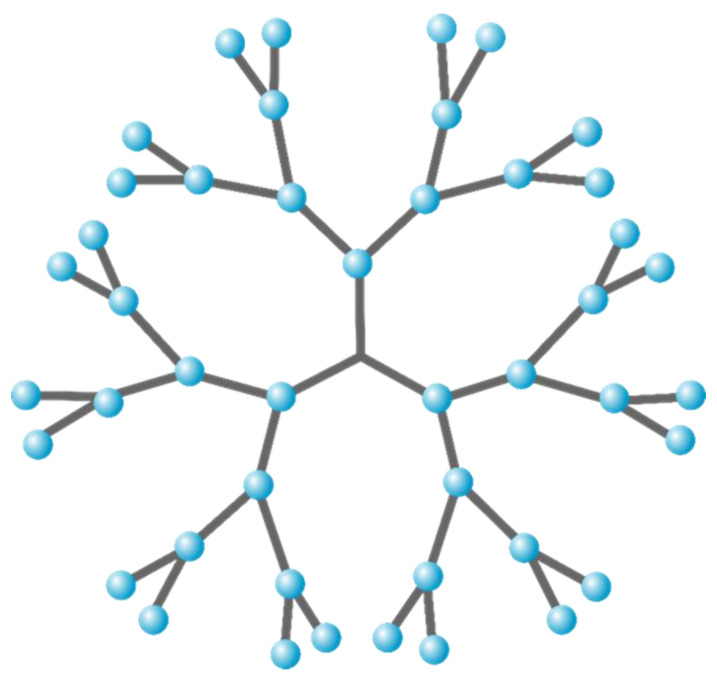
Dendritic hyperbranched polyol polyester structure.

Hyperbranched polyol polyesters include both macromolecular structures and dendrimers. Dendrimers are a class of highly branched monodisperse macromolecules having regular cascading structures (Figure 1). In contrast, macromolecular structures have also been designed with random hyperbranched structures with highly disperse molecular weight distributions along with structural defects. Hyperbranched polymers contain a large number of branch points with many functional end groups. One of the earliest studies on hyperbranched polymers was by Flory, in which he describes the critical gel point of an AB_2_ type hyperbranched polymer [11]. Since then, many attempts have been undertaken to avoid gelation between difunctional groups (A_2_) and trifunctional (B_3_) monomers. By the addition of solvents, dilute reaction systems result that increase the molecular weights achieved prior to gelation [12,13,14,15,16]. Enzyme selectivity has been used to achieve relatively higher polyol polyester molecular weights with slower onset of branching while, in some cases, circumventing gelation [17,18,19]. Critical attributes of hyperbranched polyol polyesters are their high solubility and ability to install reactive functional groups at terminal units, and built-in reactive function. Their compact structure leads to relatively low solution viscosities and a molten state with little or no chain entanglements. Traditional synthetic routes to dendritic hyperbranched polyol polyesters require multiple steps. This is due to the need for protection–deprotection chemistry and purification processes that make large-scale synthesis quite difficult and inefficient. In contrast, hyperbranched polyester synthesis, without protection–deprotection chemistry, results in high molecular mass dispersity indices (Đ_M_) with heterogeneous branched structures. The Đ_M_ of hyperbranched polymers depends on the functional group conversion (p), which eventually leads to cross-linked structures composed of a large number of functional end groups. The chemical structure of polyol polyesters dramatically impacts its ability to exhibit good adhesion properties, which is believed to result from a mixture of hydrogen bonding interactions between the polymer and a substrate. 

Generally, polyol polyester synthesis occurs by the polycondensation of polyols and polyacids with or without a catalyst or solvent. The crystallinity, physical properties, degradation behaviors, and biocompatibility can often be modified by simply adjusting the monomer structure and feed ratio. Subsequent cure conditions can be used to create and/or increase the cross-link density. 

The purpose of this review is to summarize polyol polyester-based biodegradable polymers synthesized from different types of polyols and to relate the structure of selected building blocks to the resulting physical, mechanical and biological properties. The emphasis of polyol polyesters discussed will be those with strong potential for use in biomedical applications. Structural parameters are used to regulate biodegradation rates, biocompatibility, and physico-mechanical properties. Furthermore, the conversion of polyol polyester prepolymers to thermoset materials has been an important strategy in designing amorphous soft materials with low glass transition temperatures T_g_ (i.e., < 37 °C). This review will also discuss how polyol polyester structures can be adjusted to closely mimic the properties of natural tissues. 

## 2. Polyols

**Figure 2:**
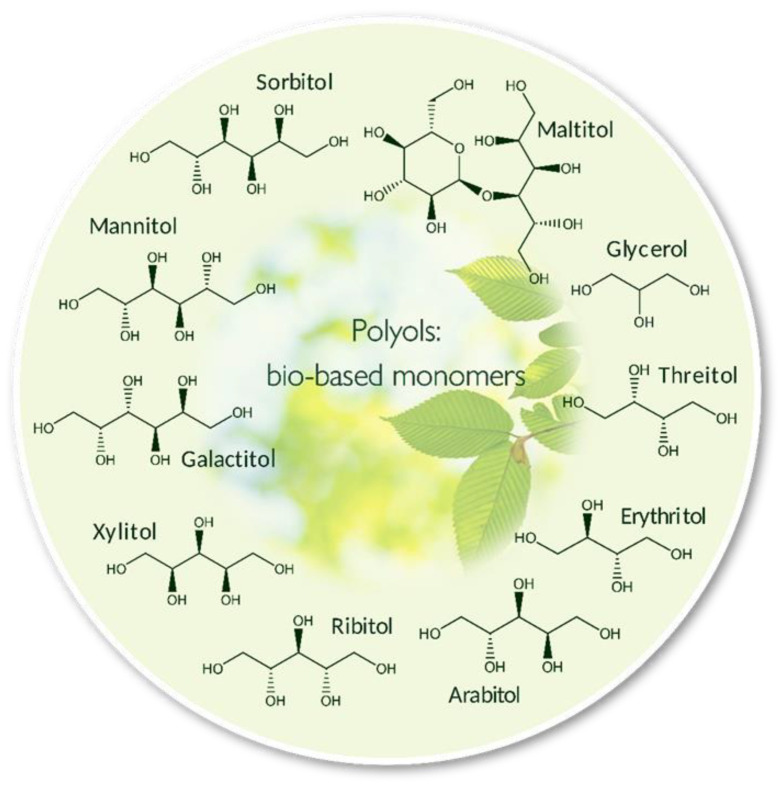
Typical polyol monomers.

A large family of polyols are sugar alcohols with variable chain length and stereochemistry (Figure 2). For example, glycerol, erythritol, and sorbitol have 3, 4, and 6 carbons and hydroxyl groups, respectively. By selecting polyols with higher chain lengths and the number of hydroxyl groups in polyol copolymerizations with diacids, the corresponding polymers formed will be decorated with a higher number of hydroxyl moieties along chains and in branches. These have been studied as polyol building blocks along with diacids for polycondensation reactions to prepare polyol polyesters. Reaction time, temperature, and pressure, as well as the ratio of each starting monomer as well as other factors can influence the properties of resulting polymers [20]. Lipase catalysis has been used for preparing high molecular weight polyol polyesters [21]. Over the past few decades, the most intensively studied enzymatic biocatalyst has been lipase B from *Candida antarctica* (CALB). Lipases require no cofactors, and they function under mild conditions that avoid side reactions such as sorbitol cyclization, and with selectivity that provides a degree of control on branch reactions and circumvents to a large extent cross-link reactions due to steric hindrance at the active site [21]. The fact that cross-link reactions occur at low frequency permits the synthesis of polyol polyesters of substantially higher molar masses than by using conventional chemical catalysts [22]. 

### 2.1. Glycerol

Glycerol, 1,2,3-propanetriol, is a by-product of large-scale biodiesel production, for which new applications are being sought. It is a simple triol with a melting temperature (T_m_) of 17.8 °C and a boiling point of 290 °C [23]. At room temperature, glycerol is a transparent, viscous, and hygroscopic liquid. It is the most commonly used bio-based monomer. Although glycerol could be burnt as a fuel, it can also be processed into high-value products. Glycerol is a fundamental component of plant oils that has received approval for medical applications by the U.S. Food and Drug Administration (FDA) [24]. As a miscellaneous and general food additive, it is generally regarded as safe (GRAS), and it is also allowed to be used in food packaging. Glycerin is found in the form of triglycerides that consist of glycerol esterified with three fatty acid molecules. Glycerol is a biocompatible monomer even at high concentrations, and it has little effect on enzymatic or metabolic processes [25]. As a result, glycerol is widely used as a cryoprotectant, where glycerol is dissolved in water and reduces freezing damage of various biomolecules [26]. Since glycerol consists of two primary and one secondary hydroxyl group, it serves as a trifunctional monomeric building for the synthesis of a wide range of hyperbranched polymers. When glycerol triol reacts with diacid monomers with non-selective catalysts, an increase in the functional group conversion ultimately leads to a three-dimensional cross-linked network. Again, without a selective catalyst, the primary hydroxyl groups are consumed more rapidly than those at the secondary position. Three hydroxyl groups of glycerol, one secondary and two primary hydroxyl groups, do not have the same reactivity [27]. For example, in the synthesis of glycerol-adipic acid polyester, the relative reactivity of each primary hydroxyl is about 3.3 times that of the secondary [28]. Typically, the diacid reacts with the primary hydroxyl groups and, to a lesser extent, with the secondary hydroxyl group. In the second step, since the concentration of primary hydroxyl groups is greatly reduced, the esterification of secondary hydroxyl groups is dominant, forming cross-links between linear polyester chains.

### 2.2. Erythritol and Threitol

In the early 1980s, a new food ingredient, erythritol, was developed by Cerestar to meet the growing consumer demand for health and calorie-controlled foods [29]. Erythritol, (2*R*,3*S*)-butane-1,2,3,4-tetrol, is a natural constituent in grapes, peaches, pears, watermelons, and mushrooms. It is a white, anhydrous, non-hygroscopic, and crystalline substance that has an appearance similar to sucrose. It has a T_g_ at −42 °C and a T_m_ of 121 °C [30]. Erythritol can be prepared from the glucose polymer, starch, through a completely biotechnological process, by combining an enzymatic conversion and fermentation. Erythritol has 2- to 4-times the digestive tolerance of other polyols, such as sorbitol, mannitol, xylitol, or glycerol. It is approved for use in foods in many countries, and the list keeps growing as does its range of applications [29]. Erythritol is a sweet polyol that has health benefiting properties, such as being tooth-friendly and safe for use by people with diabetes [31]. It has two additional critical nutritional advantages: a zero caloric value and an acceptable tolerance in humans. This tolerance results from its low molecular weight, which allows erythritol to be rapidly absorbed from the small intestine and then excreted in the urine. Excess erythritol can also be excreted unchanged without adversely impacting the normal metabolic pathways in humans.

Many polyol polyesters are based on erythritol which, with respect to its small molecular size and multifunctionality, provides unique characteristics. The low solubility and crystallinity of erythritol make it suitable for many commercial applications. The new commercially available chiral additives d-threitol ([2*R*,3*R*]-1,2,3,4-butanetetrol) and l-threitol ([2*S*,3*S*]-1,2,3,4-butanetetrol), are enantiomers produced from the reduction of tartaric acid. 

### 2.3. Xylitol

Xylitol, (2*R*,3*R*,4*S*)-pentane-1,2,3,4,5-pentol, is a sugar alcohol that occurs naturally in fruits and vegetables, such as lettuce, broccoli, raspberries, grapes, bananas, and strawberries. It is also found in yeast, lichen, mushrooms, and seaweed. In industry, xylitol is produced by chemical hydrogenating d-xylose to xylitol [32]. An alternative route to xylitol is by fermentation using a metabolically engineered yeast [33]. Xylitol is an intermediate product of carbohydrate metabolism, and adults produce between 5 and 15 micrograms of xylitol per day. 

### 2.4. Sorbitol, Mannitol, and Galactitol

Sorbitol ([2*S*,3*R*,4*R*,5*R*]-hexane-1,2,3,4,5,6-hexol), mannitol ([2*R*,3*R*,4*R*,5*R*]-hexane-1,2,3,4,5,6-hexol), and galactitol ([2*R*,3*S*,4*R*,5*S*]-hexane-1,2,3,4,5,6-hexol) are natural hexitols that exist in acyclic (alditol) or cyclic (cyclohexitol) forms. The most well-studied natural polysaccharide-derived hexitol is sorbitol, also known as glucitol. Industrially, sorbitol is produced by the catalytic hydrogenation of glucose or sucrose with hydrogen or nickel catalyst at high temperature [34,35,36]. It can also be produced by electrochemical reduction of dextrose under alkaline conditions [35]. In 1872, French chemists first discovered sorbitol in the berries of mountain ash. Most sorbitol is made from potato starch or cellulose, but it can also be found in nature (e.g., apples, pears, peaches, and plums). Similar to xylitol and erythritol, sorbitol has a negative heat of solution. Like many other sugar alcohols, the FDA also recognizes sorbitol as safe. It is also increasingly used as a moisturizer, softener, and anti-crystallization agent [34,37]. Sorbitol is also the basis for commodity chemicals and renewable resins. The advantage of using sorbitol as a monomer is that it is derived from an inexpensive renewable resource and has six hydroxyl groups (2 primary and 4 secondary) that, when reacted with a diacid using non-selective catalysts, at sufficiently high functional conversion, will form cross-linked networks that can be specifically adjusted to manipulate cross-link density and more. 

Mannitol is a diastereomer of sorbitol. In 1939, Madinaveitia and Orozco first reported the presence of mannitol in Atrovirens leaves. The accumulation of this hexitol is mainly observed in angiosperms, especially in the celery family. Mannitol is used as a carrier and stabilizer in tablets and as a diuretic in neuropharmacology. Mannitol is also an antioxidant, and it is claimed it protects against the development of colon cancer [38,39,40]. Galactitol, also known as dulcitol, is the reduction product of galactose. Galactitol-based polymers can be synthesized by polyesterification reactions with various dicarboxylic acids (such as adipic acid and dodecanedioic acid). They also showed good cytocompatibility and potential for use in bone tissue engineering [41].

### 2.5. Maltitol

Maltitol (4-*O*-α-d-glucopyranosyl-d-glucitol), with nine hydroxyl groups, has also been used for the synthesis of polyol polyesters. Maltitol is produced by hydrogenation of maltose, which is obtained in converting starch to maltose. Industrially, maltitol is one of the most commonly produced polyols with an approximate volume of 160,000 metric tons [42]. Maltitol has a T_g_ of 43.1 °C and a T_m_ of 149.6 °C [43]. It is a non-toxic, cytocompatible, sugar alcohol, and is an endogenous substance in human metabolism. In addition, due to the presence of its nine hydroxyl groups, it provides a polyol monomer that can be used to incorporate along chains multiple free hydroxyl groups as well as to increase the cross-link density of three-dimensional matrices.

## 3. Polyol Polyesters from Glycerol and Sugar Alcohols (Alditols)

Examples of polyol polyesters that provide a diverse family of controllable structured scaffolds are described in this section. Polyol polyesters with free functional groups can be further modified to form many different biomaterials providing improved control of biomaterial interaction with cells and tissues.

### 3.1. Glycerol-Based Polyesters

**Figure 3:**
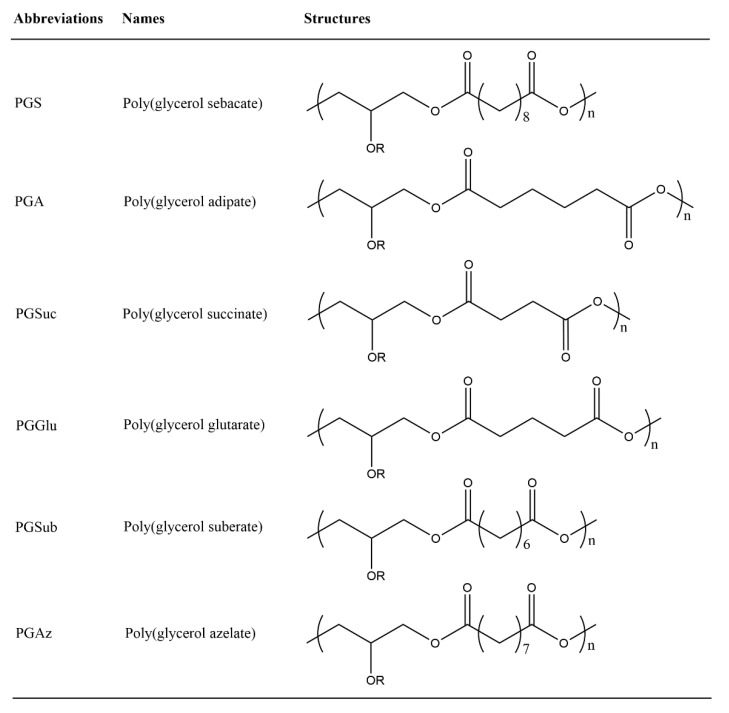
The abbreviations, names, and structures of glycerol-based polyesters.

Glycerol-based polyesters with unreacted hydroxyl groups are suitable for further functionalization after polymerization (Figure 3). Furthermore, their structural characteristics can be varied to meet performance requirements. Four major strategies have been developed to synthesize glycerol-based polyesters. The first is the epoxide ring-opening polymerization between sebacic acid and diglycidyl sebacate catalyzed by tetrabutylammonium bromide [44]. The resulting polymer has Mn and Đ values of 12.9–50.4 kDa and 1.9–8.3, respectively, after being purified by repeated precipitation [45]. The second is the synthesis of linear 1,3-linked glycerol polyesters using a catalyst like diarylborinic acid (Ar2BOH) [46]. Without further purification, the resulting polymer has an Mn and Đ of 19.2 kDa and 1.2, with only 6% of 1,2-linked glycerol polyesters. The third is the enzyme-catalyzed polycondensation reaction between glycerol and diacid/diester [19,47,48,49,50,51,52,53,54,55,56,57]. Finally, glycerol polyol polyesters were synthesized without the addition of a catalyst [58]. That is, they are self-catalyzed by acid groups present in the reaction mixture.

A prominent glyceryl polyester used in biomedical applications is polyglycerol sebacate (PGS). PGS was first reported by the Langer group in 2002 [58]. Since it is flexible as well as elastic, it has primarily been studied for the replacement of soft tissues. Examples of regenerative tissue engineering application targets include cartilage, cardiac muscle, and retina [24]. To prepare a PGS prepolymer, typically, polycondensation reactions are conducted using an equimolar concentration of glycerol and sebacic acid without solvent, maintaining the reaction temperature at 120 °C under vacuum (1 Torr) for 24 h, and then the pressure is reduced to 40 mTorr and the reaction is continued for 5 h. To promote cross-linking, the prepolymer is maintained at 120 °C under 40 mTorr for an additional two days [58]. As discussed above, the method of curing dramatically alters PGS mechanical properties. For example, Chen et al. determined the correlation between the Young’s modulus and curing temperatures. By increasing the curing temperature (110, 120, and 130 °C) the corresponding values of Young’s modulus were 0.056, 0.22, 1.2 MPa, respectively [59]. Based on these and other studies, variation in the degree of cross-linking achieved during the curing process has resulted in differences in cross-linked PGS Young’s modulus of 0.05–1.5 MPa, ultimate tensile strength values of 0.4−0.7 MPa, and a failure strain of 120−300% [60].

PGS physico-mechanical and biodegradation kinetics have also been tuned by changes in the relative concentrations of glycerol and sebacate as well as by varying other reaction parameters [60,61]. With the increase of molar ratio between glycerol: sebacic acid from 3:4 to 4:3, the tangent modulus decreases from 4.5 MPa to 1 MPa when curing for 72 h. Various composite materials have been prepared using cellulose, carbon nanotubes, and silicon as fillers to change PGS’s physical properties [62,63,64,65]. 

To increase the rate of prepolymer synthesis, Gao et al. bubbled nitrogen through the reaction mixture to accelerate the evolution of water formed [66]. The resultant polymer was a pale yellow, highly viscous liquid. Aydin et al. accelerated PGS prepolymer synthesis by a microwave-assisted process [67]. No purge gas, catalyst, or vacuum was used in the prepolymerization step. These authors reported that the degree of esterification at 130 °C in 15 min was equivalent to a conventional 6 h polymerization reaction. Tensile tests showed that the PGS elastomers synthesized via microwave-assisted process have a Young’s modulus of 0.50 ± 0.02 MPa, tensile strength of 0.27 ± 0.06 MPa, and elongation at break of 180%. The change in properties is triggered by intensive glycerol evaporation which alters glycerol to SA ratio during the prepolymerization. 

An alternative approach to PGS and PGS analog synthesis used as catalyst *Candida antarctica* lipase B (CALB) [68,69]. The CALB used is immobilized on a macroporous methyl methacrylate cross-linked resin. Most commonly, the immobilized CALB catalyst is Novozym 435 (N435), a commercial product of Novozymes. N435-catalyzed synthesis of glycerol polyesters is usually performed by a one-pot synthetic method [70,71,72]. Lang et al. described a family of poly(glycerol sebacate) (PGS) analogs where glycerol was replaced in part with octanediol (O) in bulk copolymerizations [68]. The new PGS analogs, PGOS polymers, showed tunable molecular weights with an M_n_ and M_w_ of 9500 and 92,000, as well as excellent electrospinning properties. By varying the molar ratio of glycerol to octanediol from 1:1 to 1:4, the peak melting temperature increased from 27 to 47 °C. X-ray diffraction (XRD) was also used to confirm PGOS’s ability to crystallize. Perin et al. also studied the kinetics, chain growth, and branching behavior of enzymatic synthesis of poly(glycerol sebacate) using N435 [69]; 16 kDa of mass average molecular weight and 41% of the degree of branching were achieved in acetone at 40 °C. The solvent choice impacts the solvation of the polymer coil and the structure of the enzyme, which in turn affects the degree of polymerization. CALB-catalyzed esterification reaction and acyl transfer occur simultaneously. They found out that the latter is mainly responsible for the esterification of secondary hydroxyl groups and produces branched chains.

While PGS prepolymers are most often cross-linked during the curing step by forming ester links between chains, other cross-linking strategies have been pursued. In one example, methylene diphenyl diisocyanate (MDI) was used as a cross-linking agent that reinforces Young’s modulus by adding molecules to the PGS matrix at the molecular level due to rigid phenyl groups. The resulting material showed an increase in Young’s modulus from 0.34 ± 0.04 to 1.62 ± 0.09 MPa while retaining its elasticity [73,74]. Photopolymerization methods as well as the introduction of temperature-sensitive side chain moieties have also been used for in vivo applications. In one example, a PGS prepolymer was decorated with acrylate groups and then cross-linked by ultraviolet light with a 0.1% (*w/w*) photoinitiator [75]. By changing the degree of acrylate (DA), these networks exhibit Young’s modulus of 0.05–1.38 MPa, ultimate strength of 0.05–0.50 MPa, and elongation at break of 42–189%. 

Poly(glycerol adipate) (PGA) is obtained from glycerol and adipic acid (A), and can be modified with stearic acid (S) to form PGAS. Kallinteri and coworkers synthesized PGA copolyesters in 2005 for nanoparticle drug delivery systems [54]. Depending on the catalyst and reaction conditions, PGA could be prepared in the form of linear as well as hyperbranched architectures. Under enzymatic catalysis, using an enzyme with selectivity for the primary hydroxyl groups of glycerol, the yield of the polycondensation reaction was very high (90–95%) [47]. The resulting polymer has an M_w_ range from 3–14 kDa with Đ values of 2.25–3.10. Due to steric hindrance encountered within the catalytic cleft of the enzyme, linear PGA can be obtained without the protection and deprotection process. As discussed in the PGS section, the most widely used enzymatic catalyst for PGA synthesis is N435. Many researchers have interrogated how the reaction temperature alters PGA’s degree of branching and the molecular weight synthesized by N435 catalysis [76,77]. Taresco et al. found that when the reaction temperature increased from 50 to 70 °C, the degree of branching could be increased from 5% to 30%. Without further precipitation, after 24 h polymerization, the average molecular weight of PGA decreased from 13.0 to 5.2 kDa, and Ð values increased from 2.7 to 4.2 as the temperature of synthesis increased from 50 to 70 °C.

The synthesis of the hyperbranched PGA elastomer was carried out through a catalyst-free polycondensation at 150 °C under atmospheric pressure for 17 h, followed by applying a vacuum of 3 mm Hg for 4 h [78]. PGA prepolymer was then cross-linked by heating at 120 °C for 100 h to prepare an elastomeric material. The PGA synthesized at a G/A molar ratio of 1:0.6 afforded a polymer with an Mw, Đ, and T_g_ of 4500, 2.2–2.5 °C, while PGA prepared at a G/A molar ratio of 1:1 afforded a polymer with an M_w_, Đ, and T_g_ of 12,500, 7.3, and −15 °C. Comparison of PGA synthesized with a G:A ratio of 1:1 and 1:0.6 molar ratio showed that the former has a higher Young’s modulus and tensile strength. Analysis of the thermal stability of hyperbranched PGA, prepared at a 1:1 molar ratio of G/A, showed no obvious thermal degradation up to 200–300 °C [79].

Linear PGA is an amorphous, amphiphilic, water-swellable, high-viscosity polymer synthesized by the enzymatic method mentioned above [77]. Linear PGA has shown promise as a polymer matrix for drug delivery. PGA nanoparticles (NPs) showed no hemolytic activity at concentrations up to 10 mg/mL [80]. PGA-based NPs were non-toxic to HepG2 cells. An advantage of linear PGA over PLA and PLGA is that the former can be decorated with various acyl groups, thereby changing the polymers’ hydrophobicity [80,81,82,83,84]. This is particularly valuable when considering that incorporating acyl side groups can be used to tune the hydrophilic-hydrophobic character of NP matrices to increase the loading efficiencies and thereby accommodate a diverse range of drugs [56,77,85,86,87].

Succinic acid (SuA), a white odorless solid that readily dissolves in acetone, ethanol, and water, is a highly abundant four-carbon aliphatic diacid that is available from natural sources. Both succinic acid and glycerol are classified by the FDA as safe for medical applications. Carnahan et al. first reported the synthesis of poly(glyceryl succinate) (PGSuc) dendrimers [88]. This was accomplished by adopting the protecting group benzylidene acetal that can be removed selectively and under mild conditions. All the dendrimers prepared were amorphous solids and, as the dendrimer molecular weight was increased from 1500 to 8720 (SEC molecular weights relative to polystyrene standards), their T_g_ increased from −20.2 °C to −13.5 °C [89]. Agach et al. reported the catalyst and solvent-free synthesis of PGSuc hyperbranched oligoesters with varied molecular weights and topological features [90]. Polymerizations were performed at 190 °C, under ambient pressure, for 24 h under constant mechanical stirring. End group measurements were performed as the reaction proceeded, and 93–97% of the carboxyl groups were esterified within 12 h depending on the Gly/SuA molar ratios. To increase the quantity of unreacted hydroxyl groups, higher Gly/SuA molar ratios were used to obtain M_n_ with 1116 g mol^−1^ without fractionation. All polyesters were amorphous with T_g_ values between −15.2 °C and −29.9 °C. Wyatt et al. synthesized prepolymers by reacting glycerol and succinic acid at relatively lower temperatures by using Ti(OC_4_H_9_)_4_ as catalyst [91]. Bulk polymerizations were performed using equimolar quantities of glycerol and succinic acid, under reduced pressure (~150 Pa) with 0.15% *w/w* Ti(OC_4_H_9_)_4_ catalyst. The reactants were maintained at 60 °C for 1 h, 100 °C for 2 h, 120 °C for 2 h, and 150 °C for 11 h. A hyperbranched prepolymer with an average percentage branching of 62% was obtained with no observed gelation. The M_w_, Đ, and degree of polymerization after fractionation by dissolving in methanol and passing through a column to remove any residual starting materials and catalyst, were 992 gmol^−1^, 1.28, and 3.0, respectively. The products were obtained at an average yield of 62%. These authors also reported PGSuc that was side-chain modified and synthesized with the addition of other comonomers. Baharu et al. prepared elastic copolyesters by the catalyst-free copolymerization of succinic acid, azelaic acid (C9-aliphatic diacid), and glycerol. The resulting molecular weight of poly(glycerol/azelate/succinate), PGAS increase in the following order: p(G_1_A_0.75_S_0.25_) > p(G_1_A_0.5_S_0.5_) > p(G_1_A_1_S_0_) with varying initial azelate/succinate mole ratios [92]. The PGAS copolymer was synthesized in bulk by melt polycondensation at 160–165 °C in a silicon oil bath for 2 h under a constant stream of nitrogen. The PGAS synthesized at an A/S molar ratio of 1:0 afforded a polymer with an M_n_, M_w_, and Đ of 515 g mol^−1^, 13883 g mol^−1^, 26.9, while PGAS prepared at an A/S ratio of 0.75:0.25 afforded a polymer with an M_n_, M_w_, and Đ of 3656 g mol^−1^, 24626 g mol^−1^, 6.73, respectively. The copolyester T_g_ was varied from −23 °C to −8 °C by changing the ratio of comonomers in the feed of azelate/succinate from 1:0 to 0.5:0.5. 

Compared to the aforementioned glycerol-based polyesters, poly(glycerol-*co*-suberate), poly(glycerol-*co*-azelate), and poly(glycerol-*co*-glutarate) have received less attention. Glutaric acid has been used to produce glyceryl polyester. Wyatt et al. first reported the synthesis of poly(glycerol-*co*-glutarate), PGGlu in 2010. [93] Glutaric acid (a C5 aliphatic diacid, also known as pentanedioic acid) was dissolved in dimethylformamide (DMF) or dimethyl sulfoxide (DMSO), and then the catalyst 0.15% *w/w* titanium tetroxide, glycerol, and butanol was slowly added while maintaining the reaction at 150 °C (22 h) under ambient pressure. The resulting oligoester product had an M_n_ and Đ of 2402 g mol^−1^ and 1.09, respectively. According to their reports, the T_g_ values of PGGlu prepared in toluene at 135 °Care −13.9 °C and −13.0 °C, with the G/Glu molar ratios of 1:2 and 2:1, respectively [94]. These hyperbranched polyesters had low tensile strengths (0.41 and 0.62 MPa, respectively). The values of Young’s modulus were 1.94 and 4.58 MPa, and tensile elongation at break are 26.9% and 16.3%, respectively.

To the best of our knowledge, the only report on poly(glyceryl-*co*-suberate), PGSub, is that by Unnisa et al. [95]. PGSub was synthesized by reacting glycerol and suberic acid at a ratio of 1:1 under nitrogen flow at 140 °C until the solution becomes homogeneous. The resulting prepolymer was blended with PVA with a PVA/PGsub mass ratio of 1:0.75, then cured with various amounts of NH_4_SCN (0.5, 0.6, and 0.7 g) at 60 °C until complete removal of moisture. The film has a T_g_ value of 84.54 °C for pure PGsub blend, which further increased to 116.50 °C (0.5 g), 122.35 °C (0.6 g), and 182.21 °C (0.7 g) by adding NH_4_SCN.

Wyatt and coworkers disclosed the copolymerization of glycerol and azelaic acid to prepare the hyperbranched oligomer poly(glyceryl-*co*-azelate) (PGAz) [91]. Azelaic acid and glycerin were reacted at a 1:1 molar ratio using titanium (IV) butoxide (0.15% *w/w*) as catalyst. The reaction mixture was first maintained at 60 °C under reduced pressure (~150 Pa) for 1 h. Then, the reaction temperature was increased to 150 °C gradually under reduced pressure (~150 Pa) for 16 h. The synthesized PGAz had M_n_ and Đ values of 2316 g mol^−1^ and 1.30, respectively. Kadhum et al. studied PGAz’s thermal properties with different comonomer feed ratios Az/G 1:2, 1:1, and 2:1, respectively [96]. The decomposition temperature determined by TGA was above 380 °C, where the most important decomposition occurred at ~400 °C.

### 3.2. Erythritol-Based Polyesters

A series of poly(erythritol-*co*-dicarboxylate), PErD, elastomers were synthesized without the addition of catalysts or co-reagents [97]. Polymerizations were carried out by melt polycondensation reactions with α,ω-diacids with carbon chain lengths from 5 to 14. Under an argon atmosphere, the monomer was stirred at 145 °C. After forming a homogeneous melt, the mixture was stirred for an additional 2 h. Then the pressure was reduced to 2 Torr and the material continued to be stirred for 7 h. The prepolymer was then precipitated with methanol at −78 °C. This resulted in the synthesis of poly(erythritol-*co*-adipate), PErAd; poly(erythritol-*co*-pimelate), PErPi; poly(erythritol-*co*-suberate), PErSu; poly(erythritol-*co*-azelate), PErAz; poly(erythritol-*co*-sebacate), PErSe; poly(erythritol-*co*-dodecanedioate), PErDo; and poly(erythritol tetradecanedioate), PErMyr. The prepolymers were further cured at 120 or 140 °C for 3 days. Table 1 lists T_g_ and T_m_ values determined by differential scanning calorimetry (DSC)and M_n_ and Đ values measured by gel permeation chromatography (GPC) [97]. An increase in the diacid chain length resulted in a decrease in T_g_ from −7.0 to −47 °C. Furthermore, PErSe, PErDo, and PErMyr had melting transitions at 60.2, 63.4, and 66.0 °C, respectively. By changing the chain length of the diacid, the rigidity and hydrophobicity of the resulting copolyesters could be adjusted so that the mechanical properties and degradation rate could be controlled. The molecular weight, as well as the thermal data of prepolymers are listed in Table 1. The Young’s modulus, ultimate tensile stress, and breaking strain of erythritol polyesters were 0.08–80.37 MPa, 0.14–16.65 MPa, and 22–466%, respectively. The prepolymers are compatible with imprint lithography, can be used in emulsions, and can be processed by various methods to obtain nanoparticles and embossed films. In vitro degradation studies for cured films in phosphate-buffered saline (PBS) at pH 7.4 and 37 °C showed the impact of diacid chain length for the series of PErD copolymers. PErMyr showed the slowest hydrolytic degradation (6.4% wt. loss in 6 weeks), whereas PErAd was most rapidly hydrolyzed (100% wt. loss in 3 weeks). Human mesenchymal stem cells (hMSCs) were used to study the in vitro cytotoxicity of PErDA’s in a polymer-extracted medium. Within 48 h of extract addition, cells were able to reach confluence regardless of the polymer extract used. The cell culture test with Swiss Albino 3T3 (SA) fibroblasts showed that these erythritol-based polyesters had good biocompatibility.

### 3.3. Xylitol-Based Polyesters

Xylitol-based polymers have attracted great interest as biomaterials because of their biocompatibility and biodegradability. For example, poly(xylitol sebacate) shows an in vitro and in vivo biocompatibility comparable to the widely used biodegradable polymer poly(l-lactic acid-*co*-glycolic acid), PLGA. In addition, the mechanical properties and degradation rate of xylitol-containing polyol polyester can be fine-tuned by adjusting the ratio of xylitol to dicarboxylic acid. Furthermore, the polymer properties can be further manipulated by altering the cure time as well as the dicarboxylic acid chain length. Many potential medical uses of xylitol-based polyesters have been reported. These include poly(xylitol-*co*-maleate-*co*-PEG) (PEG-Polyethylene glycol) hydrogels for cell encapsulation and poly(xylitol sebacate) fiber networks for electrospinning using the core-shell method.

**Figure 4:**
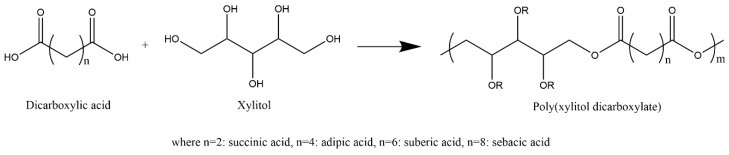
Schematic of synthesis of a series of xylitol-based polyesters [98].

In 2014, Dasgupta et al. prepared a series of xylitol-based polyesters (Figure 4) [98]. The effect of the diacid chain length (C4, C6, C8, C10) comonomer stoichiometric ratio, and post polymerization curing conditions (time, temperature) on the physicochemical properties of the corresponding copolymers was studied. The polymerization was completed by melt polycondensation at 150 °C for 2 h under a nitrogen atmosphere. Then, vacuum (−700 mmHg) was used for 14–24 h to increase the yield. The prepolymers were further cured for 3 or 14 days under −700 mmHg vacuum. The prepolymers were all white, slightly soluble in ethanol, acetone, chloroform, and more soluble in dimethylsulfoxide and dimethylformamide. The DSC data show that, as the dicarboxylic acid chain length increases, the T_g_ decreases (48 °C to 2 °C) due to increased chain rigidity associated with hydrogen bonding. They also reported that an increase in the diacid chain length results in slower xylitol-based copolyester hydrolysis. That is, with succinic and sebacic diacids, the extent of weight loss in 20 mL PBS at pH 7.4 and 37 °C in an incubator shaker at 100 rpm was 100% and 4% in seven days. Furthermore, the Young’s modulus of these xylitol-based copolyesters with succinic acid, adipic acid, suberic acid, and sebacic acid were in the range from 0.5 MPa to 15 MPa, respectively. 

Li et al. compared the properties of poly(glycerol-*co*-sebacate) (PGS) and poly(xylitol-*co*-sebacate) (PXS) [99]. The monomers reacted in a 1:1 molar ratio at 130 °C for 24 h under a nitrogen atmosphere. The prepolymers were then cured at 130 °C under vacuum for 1 to 12 days in the form of thin film. The elasticity of PXS was about twice that of their PGS analogs having the same cross-link density (~200% vs ~100%). These changes in properties were attributed to relatively longer and more orientable xylitol monomers, compared with glycerol molecules. Despite the fact that cured PXS networks should have substantially more free hydroxyl groups than that of PGS and therefore absorb more water into its network, this did not result in a more rapid enzymatic degradation of PXS when incubated at 37 °C in a tissue culture medium. However, after 35 days of in vitro incubation, the weight loss of PGS samples cross-linked at 130 °C for 7 days was on average 13%, while the weight loss of PXS samples cross-linked at 130 °C for 9 days was about 8%. This result was attributed by Li et al. to the steric hindrance of the longer polyol that protects the ester bond from attack by water molecules. An alternative explanation is that the hydrolysis of PXS is slowed since it exhibits a more extensive hydrogen bonding network that increases the difficulty of water penetration into the matrix. Thus, changing the polyol structure of polyol polyesters is a valuable tool to modulate physico-mechanical and hydrolytic behavior.

Taylor and Darin used the following equations to obtain the dependence of the elongation at break (λ_max_ = 1 +ε_max_) and UTS (Ultimate tensile strength) on the strand density:

(1) $$\lambda_{\max}=k_{1}\nu^{-0.5}$$

(2) $$\mathrm{UTS}=k_{2}\nu \;\left\{1-\frac{1}{{\left(1+k_{3}\lambda_{\max}^{3}\right)}^{0.5}}\right\}$$

where ν is the strand density, and the theoretical value of exponent n is 0.5. The constants k_1_, k_2_, and k_3_ in this equation were determined by the polymer network structure. Generally, k_1_ represents stretchability (elongation), and k_2_ represents the resistance (strength) of the network to deformation. In Li et al. work, the k_1_ and k_2_ values of the PXS network are both higher than that of the PGS, which indicates that PXS affords improved elongation properties without loss of deformation strength [99]. The theory is based on UTS being determined by the degree of strand orientation during deformation and λ_max_ being determined by the average Gaussian end-to-end distance of the network strands, proportional to the square root of the number of strands. Most importantly, under the same cross-link density, PXS is easier to stretch than PGS. The higher UTS of PXS and the better elongation at break, suggests that PXS can provide more reliable elasticity than PGS when having a similar Young’s modulus. 

### 3.4. Sorbitol-Based and Mannitol-Based Polyesters

Polyol polyesters built from sorbitol can be synthesized by acid- or base-catalyzed esterification reactions at elevated temperatures. They can also be prepared by a base-catalyzed transesterification reaction of sorbitol with fatty acid methyl esters or triglycerides. Sorbitol has the advantage of possessing a low melting point (95 °C) relative to many other alditols such as erythritol (121 °C) and mannitol (165 °C). This facilitates copolymerizations of sorbitol with diacids at relatively lower temperatures where sorbitol forms a liquid phase. As with other sugar alditols, it provides multiple hydroxyl groups to enhance hydrogen bonding interactions between chains and function as reactive sites to install various functional groups. Since base-catalyzed reactions result in undesirable, highly colored products, it is preferred to synthesize sorbitol-based polyesters by acid-catalysis. Mei et al. synthesized a family of sorbitol-based polyesters using a one-pot lipase-catalyzed polycondensation [100]. Their data demonstrated that the resulting sorbitol-containing polyester surface caused cell behavior similar to the PCL control. The monomers were mixed at 120 °C first to achieve a homogenous solution, then the temperature was decreased slowly to 95 °C, and N435 was added to the reaction. The vacuum (13.33 Pa) was applied, and 90 °C was reached for 48 h. The resulting poly(apidic acid-*co*-octanediol-*co*-sorbitol) (PAOS) was then precipitated in cold methanol/chloroform. The highest M_w_ obtained was 117 kDa with S:O:A ratio of 30:70:100, which has a Đ value of 3.4. The NIH 3T3 fibroblast cell line was chosen for the cytocompatibility study to investigate attachment, viability, proliferation, etc. The study of cell behavior indicated that different compositions of sorbitol-containing polyesters and PCL had similarly high cytocompatibility, which indicated the functional PAOS backbones could be used to develop a platform for more advanced biomaterials [100].

Fu et al. synthesized a set of poly(sorbitol adipate) and copolymers of octanediol adipate and sorbitol adipate using N435 as catalyst [101]. The reaction was performed in bulk at 90 °C under reduced pressure (40 mmHg) for 48 h. They found that with the increase of sorbitol content in the copolymer, both melting and crystallization temperatures decreased. The glass transition temperature values increased from −28 °C to 7 °C as the amount of sorbitol in the copolymer increased from 0% to 30%. The DMA data indicates that poly(sorbitol adipate) is an amorphous polymer, while all other copolymers are semicrystalline. The DSC (Differential scanning calorimetry) and WAXS (Wide-angle X-ray scattering) analysis proved that introduction of the hydrophilic polyol units in the polyester chain hinders crystallization.

Bruggeman et al. (2008) synthesized poly(sorbitol-*co*-sebacate) (PSS) by melt polycondensation without catalyst [102]. The monomer was added at 150 °C under inert gas and stirred for 2 h. Then vacuum (~50 mTorr) was applied for another 2–12 h to yield the prepolymers. This method for PSS synthesis is simple and has the advantage that products formed lack catalyst impurities. The PSS synthesized at a sorbitol/sebacate molar ratio of 1:1 afforded a polymer with an M_n_, M_w_, and Đ of 3987 g mol^−1^, 6093 g mol^−1^, 1.5, while PSS prepared at a sorbitol/sebacate ratio of 1:2 afforded a polymer with an M_n_, M_w_, and Đ of 8990 g mol^−1^, 23013 g mol^−1^, 2.6, respectively. The prepolymers were further cured at 120 °C under vacuum (~2 Pa) for 4 days. However, the tensile strength and modulus of the resulting PSS were low, limiting its application in situations requiring higher load-bearing materials. 

**Figure 5:**
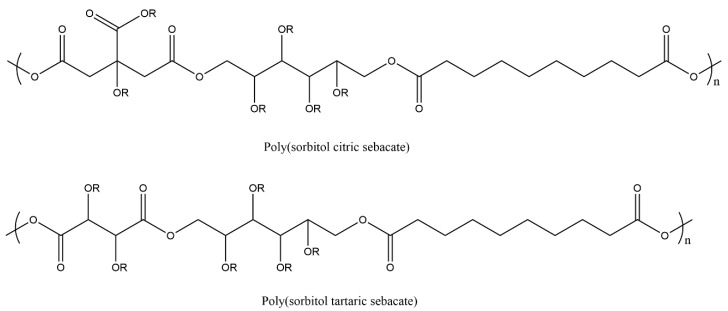
Structure of poly(sorbitol-co-citrate-*co*-sebacate) (PSCS) and poly(sorbitol-*co*-tartaric-*co*-sebacate) (PSTS) where R groups are either esters or free hydroxyl moieties.

Multifunctional monomers such as citric acid or tartaric acid have been incorporated to act as secondary cross-linkingcross-linking agents to improve the mechanical properties of PSS (Figure 5). The resulting polyesters, poly(sorbitol-*co*-citrate-*co*-sebacate), PSCS, and poly(sorbitol-*co*-tartaric-*co*-sebacate), PSTS showed significant improvements in mechanical properties [103]. The Young’s modulus, tensile strength, and elongation at break of the PSTS are 7.15 ± 0.38 MPa, 0.45 ± 0.04 MPa, 578.36 ± 51.27%, while and PSCS has a value of 462.65 ± 34.21MPa, 20.32 ± 2.54 MPa, and 25.94 ± 4.02%, respectively.

Kamaruzaman et al. synthesized poly(sorbitol-*co*-azelate) (PSAz) in bulk catalyzed by tin oxide (ii) [104]. They found that the preferred reaction conditions to achieve the highest monomer conversion are a temperature of 160 °C, sorbitol to azelaic acid molar ratio of 4:1 and 2 wt% catalyst content. After a 6 h reaction, the azelaic acid conversion reached 72%. Gustini et al. reported N435 catalyzed copolymerizations of sorbitol, 1,10-decanediol, and a series of dicarboxylic acids under solvent-free conditions [105]. The molecular weight of the corresponding polyesters was a function of the reaction time, enzyme loading, and reaction stoichiometry. The color of the polyesters obtained varied from white to yellowish with M_n_ values between 4 and 6 kg mol^−1^. Thermal analysis by DSC revealed that the copolymers are amorphous materials with T_g_ around −50 °C and T_m_ around 30 °C. The temperature for the maximum decomposition rate was between 330 °C and 410 °C.

### 3.5. Maltitol-Based Polyesters

**Figure 6:**
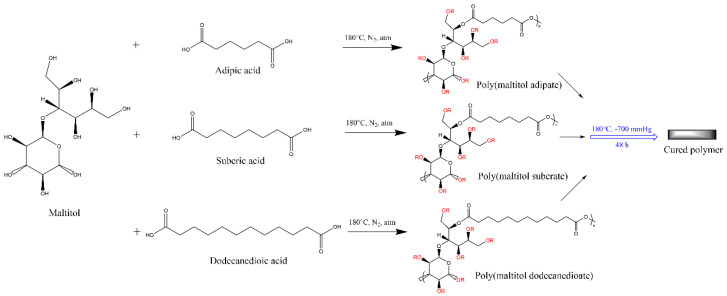
Reaction scheme for the synthesis of poly(maltitol-*co*-adipate), poly(maltitol-*co*-suberate), and poly(maltitol-*co*-dodecanedioate) where R groups are either esters or free hydroxyl moieties [106].

Natarajan et al. reacted maltitol with various dicarboxylic acids (adipic acid, dodecanedioic acid, and suberic acid) with variation in the comonomer stoichiometry (Figure 6) [106]. Prepolymer synthesis was performed by a simple melt condensation at 180 °C for 2 h with continuous nitrogen purging. A two-day post-polymerization process was then conducted in a vacuum oven at 120 °C and 700 mm Hg. The resulting cross-linked materials were subjected to a hydrolytic degradation study in PBS (pH 7.4) at 37 °C. These studies revealed that all the maltitol-containing copolyesters follow first-order degradation kinetics measured by weight loss measurements as a function of time. Both degradation and dye release studies using the above described series of copolyesters had slower degradation and dye release kinetics with increased diacid chain length. Also, Natarajan et al. disclosed that hydrolytic degradation and dye release rate increase when the molar ratio of maltitol: diacid increased. In vitro cytocompatibility studies conducted using MC3T3-E1 cells are promising as they indicate the copolyesters are cytocompatible. Studies on these maltitol-based biodegradable polyesters show good cell compatibility that is suitable for bone tissue engineering.

### 3.6. Cross-Linking Polyol polyesters

The control over the degree of cross-linking and the proper selection of cross-linkers is critical in synthesizing structurally tailored polyesters scaffolds that meet specific mechanical properties, i.e., Young’s modulus, ultimate tensile strength and elongation at break, degradation and swelling behaviors required for an intended tissue engineering application [107,108]. Moreover, the selection of long polyol building blocks offers a path for cross-linked polyester networks having a high number of free hydroxyl groups that can be used for chemical functionalization [109,110]. 

Cross-linking of polyol polyesters can be achieved by the polycondensation reaction of the pendant carboxyl and hydroxyl groups of the prepolymer chains in absence of catalyst, in a process known as thermal cross-linking, and usually involves high temperatures (>100 °C), under vacuum for prolonged curing times [41,111]. Tuning of the mechanical properties of the cross-linked network can be achieved by modifying the processing conditions, albeit prepolymer structure also determines the probability and extent of cross-linking [59,111,112]. Unfortunately, given the harsh processing conditions typically employed, thermal cross-linking poses the following disadvantages: *i*) a lack of built in functional sites for bioconjugation in the resulting thermoset polyester, and *ii*) an inability to cross-link in situ in tissue or to incorporate viable cells and thermally sensitive molecules such as drugs and proteins, features that are highly sought after when designing biomaterials.

This section describes the main strategies proposed to date to overcome the limitations of cross-linking. The discussion is centered on PGS-based materials to illustrate these strategies. PGS is one of the most well-studied polyol polyesters and allows a clear picture of the impact that prepolymer structure and degree of cross-linking have on the physical properties of the resulting cross-linked networks. These properties can be varied to suit a wide range of applications for a particular prepolymer.

One alternative to thermal cross-linking is photo-induced cross-linking [113]. Incorporation of ultraviolet (UV) sensitive units along chains or as end chain groups by direct polymerization of unsaturated building blocks (e.g., itaconic acid) as well as UV-sensitive moieties such as acrylates and methacrylates through modification of the terminal and pendant hydroxyl groups of polyol polyester prepolymers, enables a more fine-grained level of control of cross-linking, and consequently of the mechanical properties [110,114,115,116,117,118,119,120,121]. By this approach, it is possible to prepare cross-linked polyester 3D networks under mild conditions and with significantly faster curing times (minutes versus days) upon exposure to UV light. For example, Nijst et al. first synthesized a photocurable PGS-derived elastomer, poly(glycerol sebacate) acrylate (PGSA), by the chemical modification of PGS prepolymers (M_n_ = 6500 g mol^−1^, M_w_ = 23,000 g mol^−1^, and Ð = 3.5) with acrylate moieties derived from the acyl substitution reaction between acryloyl chloride and the hydroxyl groups of PGS prepolymer [75]. PGS prepolymers were prepared at an equimolar ratio of glycerol and sebacic acid and used without further purification. Acryloyl chloride reacts preferentially with the hydroxyl groups of glycerol rather than with the carboxyl groups of sebacic acid. PGSA with degrees of acrylation (DA) from 0.17 to 0.54 were obtained by modifying the acryloyl chloride: hydroxyl groups ratio in the feed and further cured at room temperature via free-radical mechanism after adding a photoinitiator and exposing it to UV light for 10 min. The Young’s modulus and ultimate tensile strength of the resulting PGSA were linearly proportional to DA, with values ranging from 0.05 MPa (DA = 0.17) to 1.38 MPa (DA = 0.54) and from 0.05 MPa to 0.50 MPa, respectively. Although the Young’s modulus obtained by this method falls within the typical range of thermally cured PGS, taking advantage of the presence of UV reactive groups along the polyester chains in PGSA, the authors were able to widen Young’s modulus, ultimate strength, and elongation ranges by its copolymerization with poly(ethylene glycol) diacrylate macromers (700 g mol^−1^). By increasing the concentration of PEG diacrylate, the Young’s modulus could be adjusted to range from 0.6 to 20 MPa, the elongation from 60% to 4%, and the ultimate strength from 0.27 MPa to 0.89 MPa. The degradation rate of photocured PGSA was dependent on DA, and photocured PGSA-PEG acrylate copolymers showed a slower in vitro degradation rate compared to photo-cured PGSA (DA = 0.31) and even slower compared to thermally cross-linked PGS. This can be explained by the presence of alkyl cross-links in PGSA-PEG and PGSA in contrast to thermally cross-linked PGS, in which degradation is facilitated through hydrolysis of the ester bonds. 

Building on this work, Burdick and co-workers prepared PGSA and studied the relationship between the molecular weight and percentage of acrylation of prepolymers, and the bulk properties of the resulting photo-crosslinked networks [116]. The mechanical properties could be tuned by adjusting both the percentage of acrylation (9% to 88%) and the molecular weight of the prepolymers (M_w_ from 4000 g mol^−1^ to 23,500 g mol^−1^). An increase in the percentage of acrylation led to an increase of the Young’s modulus and a decrease of the elongation at break as a result of a greater number of cross-link sites formed. For a given percentage of acrylation, higher molecular weights resulted in similar Young’s modulus values, but more elastomeric-like features. The Young’s modulus range for photocured PGSA was wider than that reported by Nijst, varying from 0.15 MPa up to 30 MPa, expanding the range of applications from elastic and soft tissue (e.g., cardiac tissue) to less elastic and stiff tissue (e.g., bone tissue) applications. Moreover, the authors fabricated complex fibrous scaffolds from PGSA prepolymers by electrospinning using PEO or gelatin as carrier polymers, and later using extrusion-based 3D printing techniques, which would otherwise be difficult to obtain by thermal cross-linking and that better resemble features of natural tissues [116,117,118]. A small series of PGSA prepolymers of constant M_w_ (24.24 kDa), but variable percentage of acrylation (1, 14, and 23.5%) was synthesized and processed into fibrous scaffolds by electrospinning using gelatin as a carrier polymer. The mechanical properties of bulk PGSA and PGSA/gelatin scaffolds were then evaluated. The PGSA/gelatin scaffolds were also evaluated in the hydrated state. Fibrous hydrated scaffolds exhibited a similar trend in Young’s modulus to bulk samples, increasing with an increased percentage of acrylation in PGS prepolymers. From the measurements, it was not possible to conclude how the degree of acrylation influenced the mechanical performance of the scaffolds under more physiological conditions. In vitro degradation of bulk and fibrous scaffolds was monitored over 18 weeks and showed the usual correlation with the degree of acrylation. 

Recently, Wang and co-workers formulated PGSA inks suitable for digital light processing 3D printing [122]. The fabrication method that provides high scaffold resolution and processing speed. The authors investigated the impact of the prepolymer degree of acrylation (17%, 35%, and 75%) on the mechanical properties of photo-crosslinked PGSA after curing for one minute. Again, and as expected, the Young’s modulus increased with the degree of acrylation, while the elongation at break decreased.

Analogous to the incorporation of acrylate groups, norbornene groups have also been introduced to PGS (Nor-PGS) by reacting hydroxyl groups of PGS with 5-norbornene-2-carbonyl chloride [123]. In this case, the resulting Nor-PGS prepolymers were photo-crosslinked by a thiol-ene reaction of norbornene groups on PGS with a four-arm thiolated cross-linker in the presence of photoinitiator and UV light and processed into porous scaffolds by 3D-printer. Other photocurable PGS-based polyesters have also been synthesized by incorporating methacrylate groups by functionalization of the hydroxyl groups with methacrylic anhydride [121].

The curing conditions control the mechanical properties and the degradation behavior. In free radical photocuring, the formation of aliphatic backbones, the toxicity of photoinitiators and residual acrylates and methacrylates, are major limitations in developing biodegradable and biocompatible photocurable polyesters [124,125,126,127]. Therefore, alternatives inspired by naturally occurring photosensitive cross-linkers are appealing since these compounds additionally exhibit reversible photocuring (e.g., cinnamate and coumarin groups) and allow controlled degradation [128,129,130]. For example, Zhu et al. synthesized photocurable PGS-based elastomers with pendant cinnamate groups, poly(glycerol-co-sebacate)-cinnamate (PGS-CinA), by modification of PGS prepolymers with cinnamoyl chloride [131]. Cinnamate groups undergo reversible photo-dimerization by a [2 + 2] cycloaddition mechanism under UV irradiation at λ > 260 nm. The two cinnamate groups combine to form a cyclobutane ring, which at λ < 260 nm undergoes breaking and reverses to C=C double bonds [130]. The polycondensation reaction of PGS prepolymers was terminated at times prior to the gel point to obtain prepolymers (M_n_ = 3900 g mol^−1^, Ð = 1.93) with a high population of free hydroxyl groups, to further prepare PGS-CinA prepolymers with degrees of substitution (DB) ranging from 26% to 45%. It is noteworthy that DB was determined from the ratio of the proton nuclear magnetic resonance (NMR) peaks integrals of methylene groups from sebacic acid and vinylene groups from cinnamate groups, under the assumption of non-branched PGS chains. The Young’s modulus of photocured PGS-CinA ranged from 0.05 to 0.15 MPa, while the ultimate tensile strain from 80% to 140% as DS was increased from 26% to 45% with a curing time of 4 h. Furthermore, the incorporation of cinnamate groups allowed the processing of polyester elastomers into microfilms by replica molding on patterned silicon substrates after 2 h under UVB in the absence of photoinitiator.

Another alternative to preparing cross-linked polyol polyester networks is through the formation of urethane linkages between the free hydroxyl groups of prepolymers and diisocyanate cross-linkers [132,133]. Pereira et al. prepared poly(glycerol sebacate urethane) (PGSU) from PGS prepolymers (M_w_ = 12700 g mol^−1^, 4.5) using hexamethylene diisocyanate (HDI) in presence of Sn(II) 2-ethyhexanoate as catalyst at 37 °C and relatively long curing times (36 h) [132]. The authors synthesized PGSU using different cross-linker contents (1, 0.5, and 0.3 HDI to 1 PGS molar ratio), either in the presence or absence of solvent, and calculated the corresponding cross-link densities. The Young’s modulus range of films, varying from 0.1 MPa to 20 MPa, was tailored by changing the amount of urethane groups. Degradation tests were performed in vitro in the presence of cholesterol esterase and, surprisingly, showed a higher degree of cross-linking resulting in slower the degradation rate.

The mechanical properties and degradation behavior strongly depend on the degree of cross-linking since it dictates the number of cross-links formed in the polymeric network. Tuning any of these properties requires a careful balance to obtain a material that is not only mechanically strong and flexible, but also exhibits the desired degradation kinetics.

The cross-linking strategies described throughout this section broaden the processing capabilities of polyester thermosets beyond thermal cross-linking, from electrospinning to more sophisticated techniques such as soft lithography and digital light 3D printing, enabling fabrication of complex 3D scaffold morphologies. However, all of these rely on the use of toxic compounds, such as photoinitiators, chemical functionalizers, catalysts, cross-linkers, and solvents. This makes it impossible to employ some of these strategies for in situ tissue regeneration applications. In the fabrication of body implants, traces of these compounds must completely be removed to avoid potential toxicity risks. Moreover, attention should be paid to curing times in case of photo-induced cross-linking, since long UV light exposure may also limit the incorporation of cells and proteins into scaffolds regardless of low curing temperatures. There is still plenty of room left to develop cross-linking methods that cover most of the expectations from the synthetic point of view, such as the use of safe compounds, mild reaction conditions and short reaction times, that are scalable and can be performed in few reaction steps.

## 4. Conclusions

### 4.1. Physicochemical Properties

Compared with their linear counterparts of comparable molecular weight, hyperbranched polyesters exhibit a large number of terminal groups, higher solubility, and lower viscosity. Structural characterization of polyol polyesters normally includes molecular weight analysis of prepolymers by GPC, DSC, Fourier-transform infrared spectroscopy (FTIR), and nuclear magnetic resonance (1 and 2-D ^1^H NMR and ^13^C NMR experiments) spectroscopy. The latter is used to differentiate linear verse dendritic repeat units as well as end group structure. Below are selected examples that elaborate on the structural and physico-mechanical properties studies that are discussed above. 

Bruggeman et al. studied a series of poly(polyol-*co*-sebacate) copolymers [102]. The polyols included xylitol, sorbitol, mannitol, and maltitol. Also, the comonomer stoichiometry was varied. All FTIR spectra showed a stretch at 1740 cm^−1^, which corresponds to the ester bonds. A broad stretch is also observed at ~ 3448 cm^−1^, which corresponds to the hydrogen-bonded hydroxyl moieties. All the prepolymers synthesized were waxy, opaque solids at room temperature and became a clear, viscous liquid at 130 °C. The authors reported that an increase in the sebacic:polyol molar ratio in the feed resulted in a higher cross-link density and lower polymer wettability, characterized by the equilibrium hydration weight difference and measurements of contact angle. Poly(maltitol-*co*-sebacate), synthesized at a maltitol to sebacic acid feed ratio of 1:4, is a stiff material with Young’s modulus, UTS and elongation to break of 378 ± 33.0 MPa, 17.6 ± 1.30 MPa, and 10.90 ± 1.37% [102]. In contrast, poly(sorbitol-*co*-sebacate), PSS, synthesized at a sebacic-to-maltitol feed ratio of 1:1, is the softest of the copolyesters series synthesized. Its tensile Young’s modulus, UTS, and elongation at break are 0.37 ± 0.08 MPa, 0.57 ± 0.15 MPa, and 192.24 ± 60.12%. At the same molar ratio and reaction conditions, poly(xylitol-*co*-sebacate) (PXS) has a Young’s modulus and elongation at break of 0.82 ± 0.15 MPa (2.2-fold larger than PSS) and 205.16 ± 55.76%, respectively. However, the elongation at break of PSS 1:2 is larger than that of PXS 1:2, 65.94 ± 24.87% and 33.12 ± 4.85%, respectively. This apparent contradiction may be due to differences in the network cross-linking structure which should be carefully defined. 

In Barrett’s study, several elastomers were synthesized by melt-condensation reactions using erythritol as the alditol and one of eight dicarboxylic acids: adipic acid, pimelic acid, suberic acid, azelaic acid, sebacic acid, dodecanedioic acid, and tetradecanedioic acid [97]. They reported that cured materials were semicrystalline or amorphous, depending on the diacid chain length. Relatively shorter diacids (<10 carbon atoms) copolymerized with erythritol form amorphous prepolymers; while copolymers with sebacic, dodecanedioic, and tetradecanedioic acids result in semi-solid and opaque materials at room temperature. By changing the length of the diacid, the rigidity and hydrophobicity of the resulting polymer can be adjusted so that the mechanical properties and degradation rate can be controlled. The ^1^H NMR spectra of the prepolymer series had similar peaks with differences due to the position of methylene group signals. These prepolymers also show a trend of increasing M_n_ when erythritol is reacted with longer chain diacids (Table 1).

A comparative study by Li et al. measured mass loss during propagation that results from water evolution, providing an approach to determine functional group conversion [99]. The authors also performed an acid titration for PGS and PXS synthesized under the same conditions. In addition to water, they also found that glycerol evolution occurred to a greater extent than xylitol evaporation. This is important since it points out that loss of polyol by evaporation during polycondensation reactions can occur, resulting in product repeat unit compositions that differ from the feed ratio used. 

Results consistently show that the thermal stability of hyperbranched polyols increases with a higher number of functional groups [102,134,135]. Since hyperbranched polyols have a large number of hydroxyl groups, the increase in their thermal durability is attributed to enhanced intramolecular/intermolecular bridges between hydroxyl groups. 

### 4.2. Mechanical Properties

Several groups have investigated how the mechanical properties of polyol polyesters are impacted by the comonomer stoichiometry, starting monomer structures, synthetic methods used for prepolymers and the extent of cross-linking that results from curing. Furthermore, the mechanical properties of polyol polyesters are dependent on the crystallinity and sol content. While mechanical properties such as Young’s modulus and UTS are lower with increased sol content, the sol content determines the thermoformability of the cross-linked polymers. One potential method to improve the stretchability of polyol polyesters is to use relatively long polyol diacid monomers that can separate the cross-links, thereby enhancing the flexibility of the polymer network. Another potential method to enhance the elongation at break of polyol polyesters is to use longer polyol monomers that can separate cross-links, increasing the flexibility of the polymer networks compared to short polyol counterparts. The mechanical properties of polyesters are normally determined by the degree of polymerization and cross-linking, and these properties correspond to the degree of polymer esterification. Long polyol monomers are considered those with more than three hydroxyl groups. Therefore, the influence of additional hydroxyl groups on the hydrolysis of polyol polyester networks represents an additional variable. The content of hydroxyl groups can enhance water absorption within the network; however, these larger hydroxyl groups can also create strong hydrogen bonding networks that hinder network ester bond hydrolysis. Therefore, to tune polyol polyester mechanical properties and hydrolytic degradation rates, one must balance diacid length, polyol length, cross-linking extent, and crystallinity. 

### 4.3. Degradation Bbehavior

There are different types of polymer degradation, such as photodegradation, thermal degradation, mechanical degradation, and chemical degradation [136,137]. The degradability of biopolyesters was intensively studied in the early 1970s. Since then, studies have been conducted in vivo on different polyesters in the form of microspheres, nanospheres, films and implants [138]. Biodegradable polymers all contain hydrolyzable bonds, therefore, the most important degradation mechanism they exhibit is chemical degradation or enzymatic hydrolysis. Several factors affect the chemical degradation rate: the type of chemical bond, pH, copolymer composition, and water uptake. According to Barret et al., the degradation rate of polyesters is controlled by two major factors: hydrophobicity of the elastomer and the degree that the polymer network is cross-linked [97]. The degradation rates of hyperbranched polyol polyesters are generally faster than other polyesters, including polylactides and polyglycolides. Fast-degrading polyol polyester materials are generally soft and hydrophilic, whereas higher T_g_ hydrophobic materials survive longer under hydrolysis conditions. This behavior has been reported by several groups [97,139]. During the degradation process, the crystallinity increases, which is caused by preferential hydrolysis in the amorphous domain of polyester [140].

In 1977, Tokiwa and Suzuki et al. reported that isolated enzymes could attack synthetic polymers [141]. Enzymes (lipase) catalyze the hydrolysis of the ester bonds of polyesters, resulting in a reduction in chain length and ultimately a water-soluble intermediate [142]. The enzymatic depolymerization of polyesters and the ester cleavage of oligomers are affected by different factors. One of them is the necessity of long polymer chains being able to penetrate into the enzyme-active site, which makes the chain mobility a critical factor in controlling the degradation rate. As predicted by Marten et al., as the test temperature increases, the degradation rate of the semi-crystalline polyester film increases significantly [143]. Since the polymer chain mobility is theoretically correlated to the crystallinity and melting temperature, it is possible to predict and compare the degradation behavior via these parameters. The biodegradation of polymers is a surface erosion process, and the degradation rate depends on the surface area of the polymer [144]. Therefore, the increase in the surface area is a feasible method to accelerate the degradation rate, for example, creating the porous scaffold for the polyester or using nanoparticles. 

### 4.4. Biocompatibility

Generally, polymers belonging to the poly(polyol dicarboxylate) family show both in vivo and in vitro biocompatibility [102]. The toxicity of polyol polyesters results from unreacted and hydrolysis-generated carboxylic acid groups. This is similarly observed for polyesters such as PLA and its copolymers with glycolic acid. Longer curing time can improve compatibility due to a higher cross-link density that converts many of the carboxyl groups to esters and slows the generation of carboxylic acid small molecules generated during extended incubation conditions [139].

### 4.5. Summary

The lack of tough biodegradable elastomers underlies one of the most complicated and intriguing challenges in modern bioengineering, the search for affordable and effective mechanical substitutes for tissues. The efforts have included studies of bio-based polyol polyesters owing to their diversity in polymer compositions, structures, and corresponding physicochemical and biological properties. 

Over the past few decades, multiple polyol polyester architectures have been studied, however, while much progress has been made, relationships between monomer selection, the synthetic method used (including curing) and polyester properties remain unclear. Despite the multitude of studies in this area, the search for the ideal biomaterial for tissue engineering remains a challenging task. The research community has increasingly looked towards polyol polyesters that provide numerous options for tunability while retaining biocompatibility and degradation as safe products. In conclusion, we continue to witness groundbreaking developments in material science. It is expected that these developments, coupled with recent major progress in polyol polyester synthesis and ongoing fundamental research on the mechanism of biodegradation, should lead to the development of scalable, high-performance biomaterials that will undoubtedly revolutionize biomedical devices built for in vivo applications.

## Figures and Tables

**Table 1:** Molecular weight, T_g_, T_m_, and Đ values for the series of poly(erythritol dicarboxylate) prepolymers [97].

Prepolymer	M_n_^a^/g mol^−1^	T_g_(T_m_) ^b^/ °C	Đ ^a^
Poly(erythritol glutarate)	710	−7.0	1.4
Poly(erythritol adipate)	810	−15.7	1.1
Poly(erythritol pimelate)	790	−18.1	1.4
Poly(erythritol suberate)	860	−21.4	1.2
Poly(erythritol azelate)	820	−23.6	1.5
Poly(erythritol sebacate)	1020	−36.9 (60.2)	1.6
Poly(erythritol dodecanedioate)	1470	−41.5 (63.4)	1.9
Poly(erythritol tetradecanedioate)	1450	−46.8 (66.0)	2.2

^a^ Determined by GPC. ^b^ Determined by DSC.

## References

[1] Doppalapudi S., Jain A., Domb A.J., Khan W. (2016). Biodegradable polymers for targeted delivery of anti-cancer drugs. Expert Opin. Drug Deliv..

[2] Shah T.V., Vasava D.V. (2019). A glimpse of biodegradable polymers and their biomedical applications. e-Polymers.

[3] Asghari F., Samiei M., Adibkia K., Akbarzadeh A., Davaran S. (2017). Biodegradable and biocompatible polymers for tissue engineering application: A review. Artif. Cells, Nanomed. Biotechnol..

[4] Prajapati S.K., Jain A., Jain A., Jain S. (2019). Biodegradable polymers and constructs: A novel approach in drug delivery. Eur. Polym. J..

[5] Pavlath A.E. (2020). Biodegradable polymers: Why, what, how?. Phys. Sci. Rev..

[6] Ye H., Zhang K., Kai D., Li Z., Loh X.J. (2018). Polyester elastomers for soft tissue engineering. Chem. Soc. Rev..

[7] Tham W.H., Wahit M.U., Kadir M.R.A., Wong T.W., Hassan O. (2016). Polyol-based biodegradable polyesters: A short review. Rev. Chem. Eng..

[8] Bîrcă A., Gherasim O., Grumezescu V., Grumezescu A.M. (2019). Introduction in thermoplastic and thermosetting polymers. Materials for Biomedical Engineering.

[9] Grumezescu V., Grumezescu A. (2019). Thermoset and Thermoplastic Polymers. Materials for Biomedical Engineering.

[10] Vengatesan M., Varghese A., Mittal V. (2018). Thermal properties of thermoset polymers. Thermosets.

[11] Flory P.J. (1952). Molecular size distribution in three dimensional polymers. VI. Branched polymers containing A—R—Bf-1 type units. J. Am. Chem. Soc..

[12] Linko Y.-Y., Wang Z.-L., Seppälä J. (1995). Lipase-catalyzed linear aliphatic polyester synthesis in organic solvent. Enzym. Microb. Technol..

[13] Lin Q., Long T.E. (2003). Polymerization of A2 with B3 monomers: A facile approach to hyperbranched poly (aryl ester) s. Macromolecules.

[14] Stumbé J.F., Bruchmann B. (2004). Hyperbranched polyesters based on adipic acid and glycerol. Macromol. Rapid Commun..

[15] Jikei M., Kakimoto M.-A. (2001). Hyperbranched aromatic polyamides prepared by direct polycondensation. High Perform. Polym..

[16] Fang J., Kita H., Okamoto K.-i. (2000). Hyperbranched polyimides for gas separation applications. 1. Synthesis and characterization. Macromolecules.

[17] Chen B., Hu J., Miller E.M., Xie W., Cai M., Gross R.A. (2008). *Candida antarctica* lipase B chemically immobilized on epoxy-activated micro- and nanobeads: Catalysts for polyester synthesis. Biomacromolecules.

[18] Mahapatro A., Kumar A., Gross R.A. (2004). Mild, Solvent-Free ω-Hydroxy Acid Polycondensations Catalyzed by *Candida antarctica* Lipase B. Biomacromolecules.

[19] Kumar A., Kulshrestha A.S., Gao W., Gross R.A. (2003). Versatile route to polyol polyesters by lipase catalysis. Macromolecules.

[20] Dubé M.A., Salehpour S. (2014). Applying the Principles of Green Chemistry to Polymer Production Technology. Macromol. React. Eng..

[21] Gross R.A., Ganesh M., Lu W. (2010). Enzyme-catalysis breathes new life into polyester condensation polymerizations. Trends Biotechnol..

[22] Mahapatro A., Kalra B., Kumar A., Gross R.A. (2003). Lipase-Catalyzed Polycondensations: Effect of Substrates and Solvent on Chain Formation, Dispersity, and End-Group Structure. Biomacromolecules.

[23] Lide D.R. (1994). 5. Compounds 21600-27580, Pho-Zir. Handbook of Data on Organic Compounds.

[24] Rai R., Tallawi M., Grigore A., Boccaccini A.R. (2012). Synthesis, properties and biomedical applications of poly(glycerol sebacate) (PGS): A review. Prog. Polym. Sci..

[25] Hagopian K., Ramsey J., Weindruch R. (2008). Enzymes of glycerol and glyceraldehyde metabolism in mouse liver: Effects of caloric restriction and age on activities. Biosci. Rep..

[26] Bhattacharya S. (2018). Cryoprotectants and their usage in cryopreservation process. Cryopreserv. Biotechnol. Biomed Biol. Sci..

[27] Brandner J., Birkmeier R. (1960). Relative esterifiability of the primary and secondary hydroxyl groups of glycerol. J. Am. Oil Chem. Soc..

[28] Zhang T., Howell B.A., Dumitrascu A., Martin S.J., Smith P.B. (2014). Synthesis and characterization of glycerol-adipic acid hyperbranched polyesters. Polymer.

[29] Regnat K., Mach R.L., Mach-Aigner A.R. (2018). Erythritol as sweetener—Wherefrom and whereto?. Appl. Microbiol. Biotechnol..

[30] Mitchell H. (2008). Sweeteners and Sugar Alternatives in Food Technology.

[31] Saraiva A., Carrascosa C., Raheem D., Ramos F., Raposo A. (2020). Natural Sweeteners: The Relevance of Food Naturalness for Consumers, Food Security Aspects, Sustainability and Health Impacts. Int. J. Environ. Res. Public Health.

[32] Granström T.B., Izumori K., Leisola M. (2007). A rare sugar xylitol. Part II: Biotechnological production and future applications of xylitol. Appl. Microbiol. Biotechnol..

[33] Mayer G., Kulbe K.D., Nidetzky B. (2002). Utilization of xylitol dehydrogenase in a combined microbial/enzymatic process for production of xylitol from D-glucose. Biotechnology for Fuels and Chemicals.

[34] Ortiz M.E., Bleckwedel J., Raya R.R., Mozzi F. (2013). Biotechnological and in situ food production of polyols by lactic acid bacteria. Appl. Microbiol. Biotechnol..

[35] Barbieri G., Barone C., Bhagat A., Caruso G., Conley Z.R., Parisi S. (2014). Sweet compounds in foods: Sugar alcohols. The Influence of Chemistry on New Foods and Traditional Products.

[36] Kusserow B., Schimpf S., Claus P. (2003). Hydrogenation of glucose to sorbitol over nickel and ruthenium catalysts. Adv. Synth. Catal..

[37] Jonas R., Silveira M.M. (2004). Sorbitol can be produced not only chemically but also biotechnologically. Appl. Biochem. Biotechnol..

[38] Ghoreishi S., Shahrestani R.G. (2009). Innovative strategies for engineering mannitol production. Trends Food Sci. Technol..

[39] Gaspar P., Neves A.R., Ramos A., Gasson M.J., Shearman C.A., Santos H. (2004). Engineering Lactococcus lactis for production of mannitol: High yields from food-grade strains deficient in lactate dehydrogenase and the mannitol transport system. Appl. Environ. Microbiol..

[40] Song S.H., Vieille C. (2009). Recent advances in the biological production of mannitol. Appl. Microbiol. Biotechnol..

[41] Natarajan J., Movva S., Madras G., Chatterjee K. (2017). Biodegradable galactitol based crosslinked polyesters for controlled release and bone tissue engineering. Mater. Sci. Eng. C.

[42] Saraiva A., Carrascosa C., Raheem D., Ramos F., Raposo A. (2020). Maltitol: Analytical determination methods, applications in the food industry, metabolism and health impacts. Int. J. Environ. Res. Public Health.

[43] Hadjikinova R., Marudova M. (2016). Thermal behaviour of confectionary sweeteners’ blends. Bulg. Chem. Commun.

[44] You Z., Cao H., Gao J., Shin P.H., Day B.W., Wang Y. (2010). A functionalizable polyester with free hydroxyl groups and tunable physiochemical and biological properties. Biomaterials.

[45] You Z., Bi X., Wang Y. (2012). Fine control of polyester properties via epoxide ROP using monomers carrying diverse functional groups. Macromol. Biosci..

[46] Slavko E., Taylor M.S. (2017). Catalyst-controlled polycondensation of glycerol with diacyl chlorides: Linear polyesters from a trifunctional monomer. Chem. Sci..

[47] Kline B.J., Beckman E.J., Russell A.J. (1998). One-step biocatalytic synthesis of linear polyesters with pendant hydroxyl groups. J. Am. Chem. Soc..

[48] Iglesias L.E., Fukuyama Y., Nonami H., Erra-Balsells R., Baldessari A. (1999). A simple enzymatic procedure for the synthesis of a hydroxylated polyester from glycerol and adipic acid. Biotechnol. Tech..

[49] Rao Z.K., Ni H.L., Li Y., Zhu H.Y., Liu Y., Hao J.Y. (2019). Macroscopic Scaffold Control for Lipase-Catalyzed Dendritic Polyol-Polyesters. Macromol. Chem. Phys..

[50] Zeng F., Yang X., Li D., Dai L., Zhang X., Lv Y., Wei Z. (2020). Functionalized polyesters derived from glycerol: Selective polycondensation methods toward glycerol-based polyesters by different catalysts. J. Appl. Polym. Sci..

[51] Uyama H., Inada K., Kobayashi S. (1999). Regioselective polymerization of divinyl sebacate and triols using lipase catalyst. Macromol. Rapid Commun..

[52] Uyama H., Inada K., Kobayashi S. (2001). Regioselectivity control in lipase-catalyzed polymerization of divinyl sebacate and triols. Macromol. Biosci..

[53] Kulshrestha A.S., Gao W., Gross R.A. (2005). Glycerol copolyesters: Control of branching and molecular weight using a lipase catalyst. Macromolecules.

[54] Kallinteri P., Higgins S., Hutcheon G.A., St. Pourçain C.B., Garnett M.C. (2005). Novel Functionalized Biodegradable Polymers for Nanoparticle Drug Delivery Systems. Biomacromolecules.

[55] Yang Y., Lu W., Cai J., Hou Y., Ouyang S., Xie W., Gross R.A. (2011). Poly (oleic diacid-co-glycerol): Comparison of polymer structure resulting from chemical and lipase catalysis. Macromolecules.

[56] Naolou T., Weiss V., Conrad D., Busse K., Mäder K., Kressler J. (2013). Fatty acid modified poly (glycerol adipate)-Polymeric analogues of glycerides. Tailored Polymer Architectures for Pharmaceutical and Biomedical Applications.

[57] Taresco V., Creasey R., Kennon J., Mantovani G., Alexander C., Burley J.C., Garnett M.C. (2016). Variation in structure and properties of poly (glycerol adipate) via control of chain branching during enzymatic synthesis. Polymer.

[58] Wang Y., Ameer G.A., Sheppard B.J., Langer R. (2002). A tough biodegradable elastomer. Nat. Biotechnol..

[59] Chen Q.-Z., Bismarck A., Hansen U., Junaid S., Tran M.Q., Harding S.E., Ali N.N., Boccaccini A.R. (2008). Characterisation of a soft elastomer poly (glycerol sebacate) designed to match the mechanical properties of myocardial tissue. Biomaterials.

[60] Loh X.J., Abdul Karim A., Owh C. (2015). Poly(glycerol sebacate) biomaterial: Synthesis and biomedical applications. J. Mater. Chem. B.

[61] Kemppainen J.M., Hollister S.J. (2010). Tailoring the mechanical properties of 3D-designed poly(glycerol sebacate) scaffolds for cartilage applications. J. Biomed. Mater. Res. Part A Off. J. Soc. Biomater. Jpn. Soc. Biomater.Aust. Soc. Biomater. Korean Soc. Biomater..

[62] Zhou L., He H., Jiang C., He S. (2015). Preparation and characterization of poly (glycerol sebacate)/cellulose nanocrystals elastomeric composites. J. Appl. Polym. Sci..

[63] Liu Q., Wu J., Tan T., Zhang L., Chen D., Tian W. (2009). Preparation, properties and cytotoxicity evaluation of a biodegradable polyester elastomer composite. Polym. Degrad. Stab..

[64] Gaharwar A.K., Patel A., Dolatshahi-Pirouz A., Zhang H., Rangarajan K., Iviglia G., Shin S.-R., Hussain M.A., Khademhosseini A. (2015). Elastomeric nanocomposite scaffolds made from poly(glycerol sebacate) chemically crosslinked with carbon nanotubes. Biomater. Sci..

[65] Zhao X., Wu Y., Du Y., Chen X., Lei B., Xue Y., Ma P.X. (2015). A highly bioactive and biodegradable poly (glycerol sebacate)–silica glass hybrid elastomer with tailored mechanical properties for bone tissue regeneration. J. Mater. Chem. B.

[66] Gao J., Crapo P.M., Wang Y. (2006). Macroporous elastomeric scaffolds with extensive micropores for soft tissue engineering. Tissue Eng..

[67] Aydin H.M., Salimi K., Rzayev Z.M.O., Pişkin E. (2013). Microwave-assisted rapid synthesis of poly(glycerol-sebacate) elastomers. Biomater. Sci..

[68] Lang K., Bhattacharya S., Ning Z., Sánchez-Leija R.J., Bramson M.T.K., Centore R., Corr D.T., Linhardt R.J., Gross R.A. (2020). Enzymatic Polymerization of Poly(glycerol-1,8-octanediol-sebacate): Versatile Poly(glycerol sebacate) Analogues that Form Monocomponent Biodegradable Fiber Scaffolds. Biomacromolecules.

[69] Perin G.B., Felisberti M.I. (2020). Enzymatic Synthesis of Poly(glycerol sebacate): Kinetics, Chain Growth, and Branching Behavior. Macromolecules.

[70] Yoon K.R., Hong S.-P., Kong B., Choi I.S. (2012). Polycondensation of sebacic acid with primary and secondary hydroxyl groups containing diols catalyzed by *Candida antarctica* lipase B. Synth. Commun..

[71] Cha H.-J., Park J.-B., Park S. (2019). Esterification of secondary alcohols and multi-hydroxyl compounds by *Candida antarctica* lipase B and subtilisin. Biotechnol. Bioprocess Eng..

[72] Schmid R.D., Verger R. (1998). Lipases: Interfacial enzymes with attractive applications. Angew. Chem. Int. Ed..

[73] Li X., Hong A.T.L., Naskar N., Chung H.-J. (2015). Criteria for Quick and Consistent Synthesis of Poly(glycerol sebacate) for Tailored Mechanical Properties. Biomacromolecules.

[74] Wu Y., Shi R., Chen D., Zhang L., Tian W. (2012). Nanosilica filled poly (glycerol-sebacate-citrate) elastomers with improved mechanical properties, adjustable degradability, and better biocompatibility. J. Appl. Polym. Sci..

[75] Nijst C.L.E., Bruggeman J.P., Karp J.M., Ferreira L., Zumbuehl A., Bettinger C.J., Langer R. (2007). Synthesis and Characterization of Photocurable Elastomers from Poly(glycerol-co-sebacate). Biomacromolecules.

[76] Swainson S.M., Styliari I.D., Taresco V., & Garnett M.C. (2019). Poly (glycerol adipate)(PGA), an enzymatically synthesized functionalizable polyester and versatile drug delivery carrier: A literature update. Polymers.

[77] Naolou T., Jbeily M., Scholtysek P., Kressler J. (2013). Synthesis and characterization of stearoyl modified poly (glycerol adipate) containing ATRP initiator on its backbone. Adv. Mater. Res..

[78] Navarro L., Ceaglio N., Rintoul I. (2017). Structure and properties of biocompatible poly (glycerol adipate) elastomers modified with ethylene glycol. Polym. J..

[79] Zhang T., Howell B.A., Smith P.B. (2015). Thermal degradation of glycerol/adipic acid hyperbranched poly (ester) s containing either hydroxyl or carboxyl end-groups. J. Therm. Anal. Calorim..

[80] Weiss V.M., Naolou T., Groth T., Kressler J., Mäder K. (2012). In vitro toxicity of stearoyl-poly (glycerol adipate) nanoparticles. J. Appl. Biomater. Funct. Mater..

[81] Puri S., Kallinteri P., Higgins S., Hutcheon G.A., Garnett M.C. (2008). Drug incorporation and release of water soluble drugs from novel functionalised poly (glycerol adipate) nanoparticles. J. Control. Release.

[82] Abo-zeid Y., Mantovani G., Irving W.L., Garnett M.C. (2018). Synthesis of nucleoside-boronic esters hydrophobic pro-drugs: A possible route to improve hydrophilic nucleoside drug loading into polymer nanoparticles. J. Drug Deliv. Sci. Technol..

[83] Meng W., Parker T., Kallinteri P., Walker D., Higgins S., Hutcheon G.A., Garnett M.C. (2006). Uptake and metabolism of novel biodegradable poly (glycerol-adipate) nanoparticles in DAOY monolayer. J. Control. Release.

[84] Abo-zeid Y., Urbanowicz R.A., Thomson B.J., Irving W.L., Tarr A.W., Garnett M.C. (2018). Enhanced nanoparticle uptake into virus infected cells: Could nanoparticles be useful in antiviral therapy?. Int. J. Pharm..

[85] Weiss V.M., Naolou T., Hause G., Kuntsche J., Kressler J., Mäder K. (2012). Poly (glycerol adipate)-fatty acid esters as versatile nanocarriers: From nanocubes over ellipsoids to nanospheres. J. Control. Release.

[86] Orafaei H., Kallinteri P., Huggins S., Hutcheon G., Pourcain C. (2008). Novel poly (glycerol-adipate) polymers used for nanoparticle making: A study of surface free energy. Iran. J. Pharm. Res..

[87] Taresco V., Suksiriworapong J., Creasey R., Burley J.C., Mantovani G., Alexander C., Treacher K., Booth J., Garnett M.C. (2016). Properties of acyl modified poly (glycerol-adipate) comb-like polymers and their self-assembly into nanoparticles. J. Polym. Sci. Part A Polym. Chem..

[88] Carnahan M.A., Grinstaff M.W. (2001). Synthesis and Characterization of Poly(glycerol−succinic acid) Dendrimers. Macromolecules.

[89] Carnahan M.A., Grinstaff M.W. (2006). Synthesis of generational polyester dendrimers derived from glycerol and succinic or adipic acid. Macromolecules.

[90] Agach M., Delbaere S., Marinkovic S., Estrine B., Nardello-Rataj V. (2012). Characterization, stability and ecotoxic properties of readily biodegradable branched oligoesters based on bio-sourced succinic acid and glycerol. Polym. Degrad. Stab..

[91] Wyatt V.T., Nuñez A., Foglia T.A., Marmer W.N. (2006). Synthesis of hyperbranched P poly (glycerol-diacid) oligomers. J. Am. Oil Chem. Soc..

[92] Baharu M.N., Kadhum A.A.H., Al-Amiery A.A., Mohamad A.B. (2015). Synthesis and characterization of polyesters derived from glycerol, azelaic acid, and succinic acid. Green Chem. Lett. Rev..

[93] Wyatt V.T., Strahan G.D., Nuñez A. (2010). The Lewis Acid-Catalyzed Synthesis of Hyperbranched Oligo (glycerol-diacid) s in Aprotic Polar Media. J. Am. Oil Chem. Soc..

[94] Wyatt V.T., Yadav M.P., Latona N., Liu C.-K. (2013). Thermal and mechanical properties of glycerol-based polymer films infused with plant cell wall polysaccharides. J. Biobased Mater. Bioenergy.

[95] Unnisa C.N., Chitra S., Selvasekarapandian S., Monisha S., Devi G.N., Moniha V., Hema M. (2018). Development of poly (glycerol suberate) polyester (PGS)–PVA blend polymer electrolytes with NH 4 SCN and its application. Ionics.

[96] Kadhum A.A.H., Baharu M.N., Mahmood M.H. (2011). Elastic polyesters from glycerol and azelaic acid. Adv. Mater. Res..

[97] Barrett D.G., Luo W., Yousaf M.N. (2010). Aliphatic polyester elastomers derived from erythritol and α,ω-diacids. Polym. Chem..

[98] Dasgupta Q., Chatterjee K., Madras G. (2014). Combinatorial Approach to Develop Tailored Biodegradable Poly(xylitol dicarboxylate) Polyesters. Biomacromolecules.

[99] Li Y., Huang W., Cook W.D., Chen Q. (2013). A comparative study on poly (xylitol sebacate) and poly (glycerol sebacate): Mechanical properties, biodegradation and cytocompatibility. Biomed. Mater..

[100] Mei Y., Kumar A., Gao W., Gross R., Kennedy S.B., Washburn N.R., Amis E.J., Elliott J.T. (2004). Biocompatibility of sorbitol-containing polyesters. Part I: Synthesis, surface analysis and cell response in vitro. Biomaterials.

[101] Fu H., Kulshrestha A.S., Gao W., Gross R.A., Baiardo M., Scandola M. (2003). Physical characterization of sorbitol or glycerol containing aliphatic copolyesters synthesized by lipase-catalyzed polymerization. Macromolecules.

[102] Bruggeman J.P., De Bruin B.-J., Bettinger C.J., Langer R. (2008). Biodegradable poly(polyol sebacate) polymers. Biomaterials.

[103] Pasupuleti S., Madras G. (2011). Synthesis and degradation of sorbitol-based polymers. J. Appl. Polym. Sci..

[104] Kamaruzaman M.R., Chin S.Y., Pui E.C.L., Prasetiawan H., Azizan N. (2019). Synthesis of Biobased Polyester Polyol through Esterification of Sorbitol with Azelaic Acid Catalyzed by Tin(II) Oxide: A Kinetic Modeling Study. Ind. Eng. Chem. Res..

[105] Gustini L., Lavilla C., Janssen W., Martínez de Ilarduya Sáez de Asteasu D.A., Muñoz Guerra S., Koning C. (2016). Green and selective polycondensation methods toward linear sorbitol-based polyesters: Enzymatic versus organic and metal-based catalysis. ChemSusChem (Weinheim. Print).

[106] Natarajan J., Madras G., Chatterjee K. (2016). Maltitol-based biodegradable polyesters with tailored degradation and controlled release for bone regeneration. RSC Adv..

[107] Barrett D.G., Yousaf M.N. (2009). Design and applications of biodegradable polyester tissue scaffolds based on endogenous monomers found in human metabolism. Molecules.

[108] Tillet G., Boutevin B., Ameduri B. (2011). Chemical reactions of polymer cross-linking and post-cross-linking at room and medium temperature. Prog. Polym. Sci..

[109] Pellis A., Herrero Acero E., Ferrario V., Ribitsch D., Guebitz G.M., Gardossi L. (2016). The Closure of the Cycle: Enzymatic Synthesis and Functionalization of Bio-Based Polyesters. Trends Biotechnol..

[110] Bruggeman J.P., Bettinger C.J., Nijst C.L., Kohane D.S., Langer R. (2008). Biodegradable xylitol-based polymers. Adv. Mater..

[111] Valerio O., Misra M., Mohanty A.K. (2018). Poly (glycerol-co-diacids) polyesters: From glycerol biorefinery to sustainable engineering applications, a review. ACS Sustain. Chem. Eng..

[112] Conejero-García Á., Gimeno H.R., Sáez Y.M., Vilariño-Feltrer G., Ortuño-Lizarán I., Vallés-Lluch A. (2017). Correlating synthesis parameters with physicochemical properties of poly (glycerol sebacate). Eur. Polym. J..

[113] Van Bochove B., Grijpma D.W. (2019). Photo-crosslinked synthetic biodegradable polymer networks for biomedical applications. J. Biomater. Sci. Polym. Ed..

[114] Barrett D.G., Merkel T.J., Luft J.C., Yousaf M.N. (2010). One-step syntheses of photocurable polyesters based on a renewable resource. Macromolecules.

[115] Nebioglu A., Soucek M.D. (2007). Reaction kinetics and network characterization of UV-curing polyester acrylate inorganic/organic hybrids. Eur. Polym. J..

[116] Ifkovits J.L., Padera R.F., Burdick J.A. (2008). Biodegradable and radically polymerized elastomers with enhanced processing capabilities. Biomed. Mater..

[117] Ifkovits J.L., Devlin J.J., Eng G., Martens T.P., Vunjak-Novakovic G., Burdick J.A. (2009). Biodegradable fibrous scaffolds with tunable properties formed from photo-cross-linkable poly (glycerol sebacate). ACS Appl. Mater. Interfaces.

[118] Yeh Y.-C., Highley C.B., Ouyang L., Burdick J.A. (2016). 3D printing of photocurable poly (glycerol sebacate) elastomers. Biofabrication.

[119] Gerecht S., Townsend S.A., Pressler H., Zhu H., Nijst C.L., Bruggeman J.P., Nichol J.W., Langer R. (2007). A porous photocurable elastomer for cell encapsulation and culture. Biomaterials.

[120] Wang M., Lei D., Liu Z., Chen S., Sun L., Lv Z., Huang P., Jiang Z., You Z. (2017). A poly (glycerol sebacate) based photo/thermo dual curable biodegradable and biocompatible polymer for biomedical applications. J. Biomater. Sci. Polym. Ed..

[121] Pashneh-Tala S., Owen R., Bahmaee H., Rekštytė S., Malinauskas M., Claeyssens F. (2018). Synthesis, characterization and 3D micro-structuring via 2-photon polymerization of poly (glycerol sebacate)-methacrylate—an elastomeric degradable polymer. Front. Phys..

[122] Wu Y.-L., D’Amato A.R., Yan A.M., Wang R.Q., Ding X., Wang Y. (2020). Three-Dimensional Printing of Poly (glycerol sebacate) Acrylate Scaffolds via Digital Light Processing. ACS Appl. Bio Mater..

[123] Yeh Y.-C., Ouyang L., Highley C.B., Burdick J.A. (2017). Norbornene-modified poly (glycerol sebacate) as a photocurable and biodegradable elastomer. Polym. Chem..

[124] Williams C.G., Malik A.N., Kim T.K., Manson P.N., Elisseeff J.H. (2005). Variable cytocompatibility of six cell lines with photoinitiators used for polymerizing hydrogels and cell encapsulation. Biomaterials.

[125] Greim H., Ahlers J., Bias R., Broecker B., Hollander H., Gelbke H.-P., Jacobi S., Klimisch H.-J., Mangelsdorf I., Mayr W. (1995). Assessment of structurally related chemicals: Toxicity and ecotoxicity of acrylic acid and acrylic acid alkyl esters (acrylates), methacrylic acid and methacrylic acid alkyl esters (methacrylates). Chemosphere.

[126] Zondlo F.M. (2002). Final report on the safety assessment of Acrylates Copolymer and 33 related cosmetic ingredients. Int. J. Toxicol..

[127] Freidig A.P., Verhaar H.J., Hermens J.L. (1999). Comparing the potency of chemicals with multiple modes of action in aquatic toxicology: Acute toxicity due to narcosis versus reactive toxicity of acrylic compounds. Environ. Sci. Technol..

[128] Fertier L., Koleilat H., Stemmelen M., Giani O., Joly-Duhamel C., Lapinte V., Robin J.-J. (2013). The use of renewable feedstock in UV-curable materials–A new age for polymers and green chemistry. Prog. Polym. Sci..

[129] Bednarek M., Kubisa P. (2019). Reversible networks of degradable polyesters containing weak covalent bonds. Polym. Chem..

[130] Fonseca A.C., Lima M.S., Sousa A.F., Silvestre A.J., Coelho J.F., Serra A.C. (2019). Cinnamic acid derivatives as promising building blocks for advanced polymers: Synthesis, properties and applications. Polym. Chem..

[131] Zhu C., Kustra S.R., Bettinger C.J. (2013). Photocrosslinkable biodegradable elastomers based on cinnamate-functionalized polyesters. Acta Biomater..

[132] Pereira M.J.N., Ouyang B., Sundback C.A., Lang N., Friehs I., Mureli S., Pomerantseva I., McFadden J., Mochel M.C., Mwizerwa O. (2013). A highly tunable biocompatible and multifunctional biodegradable elastomer. Adv. Mater..

[133] Frydrych M., Chen B. (2017). Fabrication, structure and properties of three-dimensional biodegradable poly (glycerol sebacate urethane) scaffolds. Polymer.

[134] Krook N.M., LeBlon C., Jedlicka S.S. (2014). In Vitro Examination of Poly (glycerol sebacate) Degradation Kinetics: Effects of Porosity and Cure Temperature. MRS Online Proc. Library Arch..

[135] Li Y. (2017). Developing Poly (Polyol Sebacate)-Based Elastomeric Biomaterials for Soft Tissue Engineering. Ph.D. Thesis.

[136] Bamford C.H. (1975). Degradation of polymers. Compr. Chem. Kinet..

[137] Grassie N. (2012). Developments in Polymer Degradation—7.

[138] Mueller R.-J. (2006). Biological degradation of synthetic polyesters—Enzymes as potential catalysts for polyester recycling. Process Biochem..

[139] Li Y., Thouas G.A., Shi H., Chen Q. (2014). Enzymatic and oxidative degradation of poly (polyol sebacate). J. Biomater. Appl..

[140] Chen D., Bei J., Wang S. (2000). Polycaprolactone microparticles and their biodegradation. Polym. Degrad. Stab..

[141] Tokiwa Y., Suzuki T. (1977). Hydrolysis of polyesters by lipases. Nature.

[142] Herzog K., Müller R.-J., Deckwer W.-D. (2006). Mechanism and kinetics of the enzymatic hydrolysis of polyester nanoparticles by lipases. Polym. Degrad. Stab..

[143] Marten E., Müller R.-J., Deckwer W.-D. (2003). Studies on the enzymatic hydrolysis of polyesters I. Low molecular mass model esters and aliphatic polyesters. Polym. Degrad. Stab..

[144] Göpferich A. (1996). Mechanisms of polymer degradation and erosion. Biomaterials.

